# The causal effect of HbA1c on white matter brain aging by two-sample Mendelian randomization analysis

**DOI:** 10.3389/fnins.2023.1335500

**Published:** 2024-01-11

**Authors:** Cheng Tian, Zhenyao Ye, Rozalina G. McCoy, Yezhi Pan, Chuan Bi, Si Gao, Yizhou Ma, Mo Chen, Jiaao Yu, Tong Lu, L. Elliot Hong, Peter Kochunov, Tianzhou Ma, Shuo Chen, Song Liu

**Affiliations:** ^1^Key Laboratory of Computing Power Network and Information Security, Ministry of Education, Shandong Computer Science Center (National Supercomputer Center in Jinan), Qilu University of Technology (Shandong Academy of Sciences), Jinan, China; ^2^Shandong Engineering Research Center of Big Data Applied Technology, Faculty of Computer Science and Technology, Qilu University of Technology (Shandong Academy of Sciences), Jinan, China; ^3^Shandong Provincial Key Laboratory of Computer Networks, Shandong Fundamental Research Center for Computer Science, Jinan, China; ^4^Maryland Psychiatric Research Center, Department of Psychiatry, School of Medicine, University of Maryland, Baltimore, MD, United States; ^5^Division of Biostatistics and Bioinformatics, Department of Epidemiology and Public Health, School of Medicine, University of Maryland, Baltimore, MD, United States; ^6^Division of Endocrinology, Diabetes, & Nutrition, Department of Medicine, School of Medicine, University of Maryland, Baltimore, MD, United States; ^7^University of Maryland Institute for Health Computing, Bethesda, MD, United States; ^8^Beth Israel Deaconess Medical Center, Harvard Medical School, Boston, MA, United States; ^9^Department of Mathematics, University of Maryland, College Park, MD, United States; ^10^Department of Epidemiology and Biostatistics, School of Public Health, University of Maryland, College Park, MD, United States

**Keywords:** brain aging, gene, HbA1c, Mendelian randomization, neuroimaging, white matter integrity

## Abstract

**Background:**

Poor glycemic control with elevated levels of hemoglobin A1c (HbA1c) is associated with increased risk of cognitive impairment, with potentially varying effects between sexes. However, the causal impact of poor glycemic control on white matter brain aging in men and women is uncertain.

**Methods:**

We used two nonoverlapping data sets from UK Biobank cohort: gene-outcome group (with neuroimaging data, (*N* = 15,193; males/females: 7,101/8,092)) and gene-exposure group (without neuroimaging data, (*N* = 279,011; males/females: 122,638/156,373)). HbA1c was considered the exposure and adjusted “brain age gap” (BAG) was calculated on fractional anisotropy (FA) obtained from brain imaging as the outcome, thereby representing the difference between predicted and chronological age. The causal effects of HbA1c on adjusted BAG were studied using the generalized inverse variance weighted (gen-IVW) and other sensitivity analysis methods, including Mendelian randomization (MR)-weighted median, MR-pleiotropy residual sum and outlier, MR-using mixture models, and leave-one-out analysis.

**Results:**

We found that for every 6.75 mmol/mol increase in HbA1c, there was an increase of 0.49 (95% CI = 0.24, 0.74; *p*-value = 1.30 × 10^−4^) years in adjusted BAG. Subgroup analyses by sex and age revealed significant causal effects of HbA1c on adjusted BAG, specifically among men aged 60–73 (*p*-value = 2.37 × 10^−8^).

**Conclusion:**

Poor glycemic control has a significant causal effect on brain aging, and is most pronounced among older men aged 60–73 years, which provides insights between glycemic control and the susceptibility to age-related neurodegenerative diseases.

## 1 Introduction

Hyperglycemia, a characteristic feature of diabetes that is associated with adverse effects on brain health, has been demonstrated in multiple studies to exert toxicity on neurons (Wium-Andersen et al., 2020). This toxicity is attributed to the generation of advanced glycation end products, which induce oxidative damage and subsequent neuronal injury resulting in cognitive impairment (Gupta et al., 2022). Moreover, hyperglycemia has been associated with structural abnormalities in the brain, especially in the hippocampus, diminished white matter microstructure, and lowered gray matter density (Sharma et al., 2020). These factors collectively contribute to an elevated risk of cognitive decline and accelerate the aging process of the brain (Crane et al., 2013). However, the degree to which poor glycemic control in diabetes is associated with accelerated white matter brain aging, and whether this differs between men and women, is unknown.

Recently, a method for estimating brain age gap (BAG) was introduced, which utilizes machine-learning techniques and magnetic resonance imaging (MRI) data to identify variations in brain aging at the individual level (Bermudez et al., 2019). The BAG is calculated based on the difference between the estimated biological age and the individual’s chronological age, serving as an indicator of how much ‘older’ or ‘younger’ the individual’s brain appears compared to their chronological age. White matter, which comprises myelinated long-distance axonal projections of neurons and glial cells, plays a significant role in brain aging (Hayakawa et al., 2007). Diffusion Tensor Imaging (DTI) is a specialized MRI technique that captures the diffusion of water molecules within brain, offering valuable information about changes in white matter structure related to normal aging (Pierpaoli and Basser, 1996). One of the commonly used microstructural measures of white matter integrity in DTI is fractional anisotropy (FA), which quantifies the overall directionality of water molecule diffusion, enabling the characterization of water molecule diffusion along white matter fiber bundles (Basser and Pierpaoli, 2011). Previous studies have demonstrated regional reductions in FA with aging across the entire brain such as the cerebral hemisphere and hippocampus (Zhang et al., 2014).

Brain aging is a complex process influenced by various risk factors, including genetic, biological, and environmental factors. Among these, hyperglycemia can cause oxidative damage to pericytes, which is crucial for the integrity and functionality of the blood–brain-barrier (Salameh et al., 2016). This hyperglycemia-induced oxidative damage has been associated with cognitive decline and the development of diseases such as diabetic retinopathy and Alzheimer’s disease (Hammes et al., 2002). However, the causal relationship between hyperglycemia and the BAG remains unknown. Understanding the impact of glycemic control on brain aging and cognitive function is necessary to inform the optimal intensity of glycemic control in older adults at risk for cognitive impairment, as current clinical guidelines advocate for relaxed glycemic targets and tolerance of elevated hemoglobin A1c (HbA1c) levels in this population (ElSayed et al., 2023). Additionally, various measures of brain aging exhibit heritability and can be linked to specific genomic regions. For instance, previous research has shown that genes *THRB* and *RARB* both related to cognitive aging (Song et al., 2023). Importantly, numerous studies have reported significant sex differences in biological aging. For example, women tend to live longer than men, which corresponds to lower biological ages as determined by molecular biomarkers (e.g., DNA methylation) (Jylhävä et al., 2017). Thus, it is important to examine the impact of hyperglycemia (manifest as elevated HbA1c levels) on brain aging separately by sex.

The Mendelian randomization (MR) approach has been developed and extensively utilized to investigate causal relationships. MR employs genetic variants as instrumental variables (IVs) to infer causal relationships between an exposure and an outcome. This approach mimics the design principles of a randomized controlled trial in an observational setting (Burgess and Thompson, 2015). To ensure the validity of MR, genetic variants must satisfy the three key assumptions (Didelez and Sheehan, 2007). Firstly, the IVs should be associated with the risk factor of interest. Secondly, they should not be associated with any confounding factors that might affect the relationship between the risk factor and the outcome. Lastly, the IVs should not have a direct effect on the outcome; their impact should only occur through their influence on the risk factor itself. Two-sample MR extends this methodology by utilizing two non-overlapping sets for gene-exposure and gene-outcome analyses. Comparing to one-sample MR, it avoids the risks associated with the “winner’s curse” phenomenon (Jiang et al., 2023) and minimizes weak instrument biases (Lawlor, 2016).

Here, we examine the causal relationship between hyperglycemia and BAG using a two-sample MR analysis. We utilize data from the UK Biobank (UKBB), a large-scale prospective cohort study. The exposure variable used in our analysis is HbA1c, which provides an assessment of the average blood glucose level over approximately 3 months. We employ a machine-learning approach using FA as a measure of white matter integrity to predict brain age and calculate BAG. We hypothesized that individuals with elevated HbA1c levels would exhibit an increased BAG, suggesting potential impairment of brain microstructure. To further explore the differences between different age and sex groups, we conduct sex-stratified and age-stratified analyses. By considering the specific needs and vulnerabilities of different groups, we can tailor interventions to effectively promote brain health and enhance cognitive function across the lifespan.

## 2 Materials and methods

### 2.1 UK biobank cohort

The UKBB is a large prospective study that recruited approximately 500,000 participants aged 37 to 73 between 2006 and 2010, collecting comprehensive genetic, clinical, and phenotypic details (Ganna and Ingelsson, 2015). We utilized glycemic data from the first assessment period (2006–2010) and neuroimaging data from the second assessment visits (2014 and after) of the UKBB to ensure data quality and minimize the impact of common loss to follow-up issues. We conducted rigorous quality control (Excluding non-European or incomplete genotype individuals: *N* = 16,455) measures and focused our analyses on European ancestry with complete genotype data.

We included adults 37–73 years old with and without type 2 diabetes (T2D). To minimize the potential confounding effects of diabetes medication use in the MR analysis, we further excluded individuals who were taking diabetes medications (e.g., metformin, glipizide, and glimepiride; Supplementary Table S1) or receiving insulin injections prior to the baseline measurement. Finally, participants with brain injury, brain cancer, and mental illness were excluded (Supplementary Table S2) to minimize the impact of those conditions on FA measurements and accurately estimate normal brain aging using the adjusted BAG (Pfefferbaum et al., 2000).

### 2.2 Hyperglycemia (exposure) phenotype

**Figure 1:**
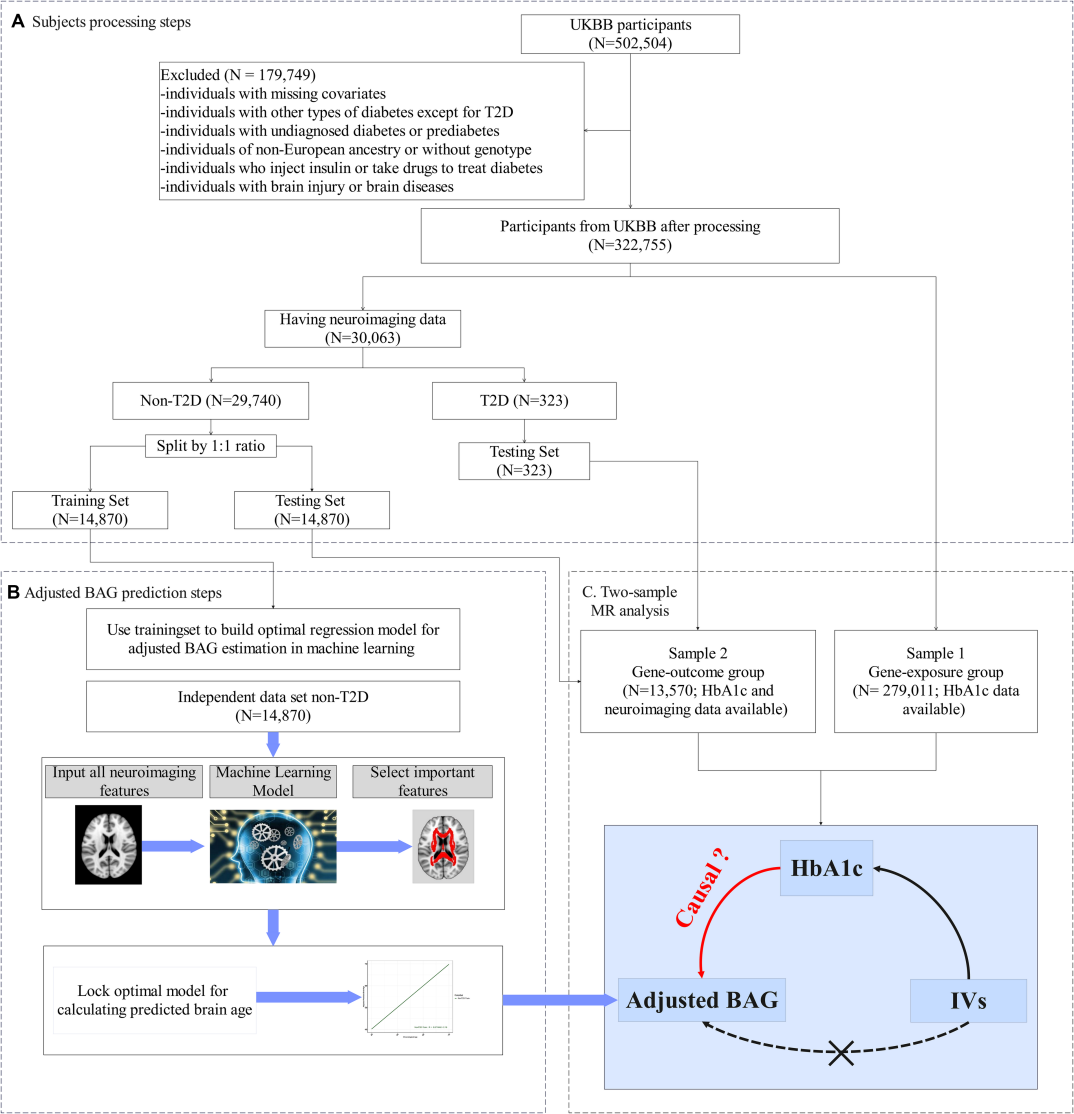
Study design. **(A)** Flowchart of our main analysis procedures and the number of subjects included at each step of the analysis. **(B)** In the training set: (i) Utilizing a machine learning algorithm to build an optimum model for predicting brain age. (ii) To minimize estimation bias, the predictive model was adjusted, yielding the adjusted predictive brain age. (iii) The outcome variable, adjusted brain age gap (BAG), was computed by subtracting the chronological age from the adjusted predictive brain age. **(C)** In the testing set, the adjusted BAG was estimated using the corrected predictive model from **(A)**. The two-sample Mendelian randomization analysis was then employed to evaluate the causal effect of HbA1c on the adjusted BAG and fractional anisotropy (FA).

This study focused on HbA1c (UKBB data field 30,750), a measure of average glycemia over approximately 3 months, as a key indicator of hyperglycemia (Sherwani et al., 2016). Individuals who self-reported a history during the first visit of type 1 diabetes, gestational diabetes, or diabetes diagnosed before the age of 18 were excluded (Peters et al., 2021). We further excluded individuals receiving glucose-lowering pharmacotherapy at the time of the first visit. Figure 1 provides a flow chart illustrating the number of participants obtained after applying the exclusion and inclusion criteria.

### 2.3 Neuroimaging data

We employed diffusion magnetic resonance imaging (dMRI) data for the 30,063 individuals who had both available genotype data and dMRI measurements (Figure 1), which were obtained through the UKBB’s imaging protocol and pipeline (Conroy et al., 2019). A total of 39 regional white matter integrity assessments as measured by FA were derived from the dMRI data, and the mean value of each white matter FA tract was assessed (see Supplementary Table S3 for more details).

### 2.4 Genotype data

UKBB utilized two genotyping chips, Affymetrix UK BiLEVE Axiom and UKBB Axiom® arrays, to capture over 90 million single nucleotide variants (SNV) from ~500,000 participants (Bycroft et al., 2017). We conducted quality controls (QCs) analysis on the genotype data, with further details on the procedures available in Supplementary Data 1. After QCs and before MR analysis, a total of 33,868 genetic variants were retained.

### 2.5 Potential confounders

We included the following variables as potential confounders in our analyses based on recommendations from previous studies (Garfield et al., 2021): age, sex, Townsend deprivation index, body mass index (BMI), systolic blood pressure, total cholesterol, smoking, triglycerides, C-reactive protein, diet, and physical activity (see Supplementary Table S4). The descriptive statistics of these variables were included in Table 1. In this study, the continuous age variable was categorized into three age groups: 37–49, 50–59, and 60–73. We conducted both overall analysis and stratified analysis by categorizing participants into sex and age subgroups.

**Table 1:** Characteristics of Participants in gene-exposure group (MR sample 1), gene-outcome group (MR sample 2 including non-T2D and T2D testing dataset) and the non-T2D training dataset (see Figure 1) from UK Biobank.

Sample distribution characteristics of all samples				
	Training Set	Gene-outcome group		gene-exposure group
		Testing Set		
	Non-T2D	Non-T2D	T2D	
Number of individuals	14,870	14,870	323	279,011
Age, mean (SD)	54.36 (7.39)	54.30 (7.41)	57.32 (7.18)	56.01 (8.11)
BMI, mean (SD)	26.30 (4.07)	26.28 (3.98)	30.69 (4.97)	27.08 (4.52)
Sex (%)				
Female	8,076 (54.31%)	7,981 (53.67%)	111 (32.41%)	156,373 (56.05%)
Male	6,794 (45.69%)	6,889 (46.33%)	212 (67.59%)	122,638 (43.95%)
Townsend deprivation index, mean(SD)	−1.95 (2.67)	−1.96 (2.68)	−1.57 (2.94)	−1.58 (2.91)
SBP, mmHg; mean (SD)	136.20 (18.70)	136.20 (18.81)	143.70 (18.99)	139.4 (19.60)
Cholesterol, mean(SD)	5.76 (1.06)	5.76 (1.06)	5.38 (1.24)	5.78 (1.10)
Smoking status (%)				
Never	10,964 (66.78%)	9,268 (62.42%)	156 (48.45%)	158,840 (57.11%)
Previous	4,667 (28.43%)	4,806 (32.37%)	147 (45.65%)	96,295 (34.62%)
Current	786 (4.79%)	773 (5.21%)	19 (5.90%)	23,017 (8.27%)
Triglyceride, mean(SD)	1.61 (0.93)	1.60 (0.93)	2.36 (1.28)	1.71 (0.99)
C Reactive Orotein, mean(SD)	1.94 (3.32)	2.00 (3.65)	3.20 (4.03)	2.39 (4.03)
Physical activities (%)				
Considered	3,429 (27.58%)	3,545 (28.33%)	66 (27.73%)	66,217 (29.44%)
Medium	9,004 (72.42%)	8,951 (71.54%)	172 (72.27%)	158,357 (70.41%)
Poor	16 (0.13%)	16 (0.13%)	0 (0.00%)	339 (0.15%)
Diet (%)				
Considered	5,242 (39.07%)	5,170 (38.31%)	108 (36.99%)	95,471 (37.97%)
Poor	8,176 (60.93%)	8,326 (61.69%)	184 (63.01%)	155,962 (62.03%)

### 2.6 Analysis overview

Our analysis comprised two steps (see Figures 1B,C). Firstly, we developed an approach to estimate adjusted BAG as our outcome variable - using machine learning techniques (e.g., random forest (RF)). This approach was implemented with FA data and chronological age, employing a training set that only included people without diabetes. During this step, we conducted 5-fold cross-validation to fine-tune the parameters and optimize the model (Figure 1B). Once the optimal model was determined, we applied it to calculate the adjusted BAG on a non-overlapping set that included individuals both with and without diabetes (i.e., testing set) (Figure 1C).

Secondly, we performed a two-sample MR analysis to examine the causal effect of HbA1c on the adjusted BAG. The first sample (referred to as the gene-exposure group) included participants with HbA1c but no neuroimaging data (*N* = 279,011). The second sample (referred to as the gene-outcome group) is consisted of distinct set of non-overlapping participants possessing both HbA1c and neuroimaging data (*N* = 13,570).

### 2.7 Adjusted BAG (outcome) computation

We firstly implemented the machine learning model with 39 FA measures to estimate a metric indicative of brain age. To identify individuals with T2D, we used the UKBB data fields 41,270 (Diagnoses- ICD10 Summary Diagnoses) and 20,002 (non-cancer illness code, self-reported medical conditions). T2D cases were defined as those having an ICD-10 code of E11-X in the UKBB data field 41,270 or having a self-reported non-cancer illness code of 1,223 for type 2 diabetes provided by UKBB data field 20,002 during the first visit (He et al., 2021). We excluded individuals without an elicited diagnosis of diabetes but with HbA1c levels >6.5% (47.5 mmol/mol) or random glucose levels >200 mg/dL during the first visit (Georgakis et al., 2021). Additionally, we excluded individuals with T2D who received glucose lowering treatment based on Supplementary Table S1.

Participants without diabetes and available neuroimaging data were randomly divided into two sets: a training set and a testing set using a 1:1 ratio (Figure 1). The training set consisted of 14,870 healthy individuals, enabling the development of an unbiased brain age prediction model applicable to the general population (Man et al., 2021). The testing set included 13,304 healthy individuals and 266 individuals with T2D who were not receiving pharmacotherapy. The descriptive statistics for both the training and testing sets are provided in Table 1.

Within training set, we employed machine learning models with an internal 5-fold cross-validation to select optimal parameters using RF. Following that, we applied recursive feature elimination (RFE) to select the most important FA measures based on their Pearson correlation coefficients (R) and mean absolute error (MAE (year)). We configured the decision trees to a count of 200, with a maximum depth of 10, a minimum branch of 5, and a minimum leaf of 2. The best predictive model was determined by comparing its minimum MAE and maximum R to those of other parametric models. Moreover, to assess the effectiveness of the RF regression method in predicting brain age, we compared some machine learning model candidates, including RF regression, gradient boosting regression (Friedman, 2002), and least absolute shrinkage and selection operator (LASSO) (Friedman et al., 2010).

Given by the optimal predictive model, we computed the predicted brain age ( $\hat{B}$ ) and then calculated the BAG ( $\mathrm{BAG}=\hat{B}-Y$ ) through the difference between the predicted brain age and the chronological age ( Y ) (Man et al., 2021). Notably, there is systematic bias in the BAG estimation, where individuals with a lower chronological age are more likely to have an overestimated BAG, while those with a higher chronological age are more likely to have an underestimated BAG (Supplementary Figure S1). To mitigate this bias, we regressed BAG on Y to adjust for the age bias based on a procedure from prior literature (Butler et al., 2021). This allowed us to obtain the adjusted predicted brain age ( ${\hat{B}}_{\mathrm{adj}}$ ) and the adjusted BAG ( ${\mathrm{BAG}}_{\mathrm{adj}}={\hat{B}}_{\mathrm{adj}}-Y$ ).

Additionally, we investigated the relationship between adjusted BAG and cognitive functions, including non-verbal reasoning (UKBB phenotype code 6333, Duration spent answering each puzzle), verbal and numerical reasoning (UKBB phenotype code 20016, Fluid intelligence score), and processing speed (UKBB phenotype code 20023, Mean time to identify matches correctly).

### 2.8 Two-sample MR analysis

We employed a two-sample MR analysis to examine the causal relationship between HbA1c on adjusted BAG. A significance level of *p* < 0.05 indicated a significant causal relationship. We conducted a GWAS analysis on Hb1Ac using individuals from the gene-exposure group (*N* = 279,011), and applied a genome-wide significant threshold (*p* < 5e-8), along with linkage disequilibrium clumping using r^2^ > 0.50 within a 1,000 kb window, to select potential IVs for the MR analysis. We further eliminated IVs associated with confounding factors in both the gene-exposure and gene-outcome groups (adjusted *p*-value >0.05 using the Benjamini-Hochberg false discovery rate method). We also removed IVs associated with our outcome variable from the gene-outcome group (N = 13,570, adjusted *p*-value >0.1). We performed a gene annotation analysis using the Functional Annotation of Variant Online Resource (FAVOR) (Zhou et al., 2023) (https://favor.genohub.org/, accessed April 26, 2023) to validate selected IVs associated with the exposure as reported in previous literature based on their functional information.

Given by selected IVs, we performed the two-sample MR analysis using generalized inverse variance weighted model (gen-IVW) (Burgess et al., 2016) through an R package ‘*Mendelian Randomization*’ (version 0.6.0) (Yavorska and Burgess, 2017). We employed Cochran’s Q test and Higgins’s I^2^ test alongside the MR analysis to assess the heterogeneity of causal effects among IVs.

We conducted a series of sensitivity analyses to enhance the robustness and reliability of our results, including Inverse variance weighted (IVW) (Bowden et al., 2015), MR-weighted-median (Bowden et al., 2016a), MR-pleiotropy residual sum and outlier (MR-PRESSO) (Verbanck et al., 2018), and MR-using mixture models (MR-MIX) (Qi and Chatterjee, 2018). MR-PRESSO (Verbanck et al., 2018) identifies and eliminates outlier IVs during the MR analysis by assessing the presence of significant horizontal pleiotropy, which leads to MR estimates with reduced variability. MR-MIX employs a mixed model to combine IVs with potential horizontal pleiotropy (Qi and Chatterjee, 2018). Additionally, we conducted a leave-one-out analysis (LOOA) to detect any potential bias introduced by individual IV and to obtain more robust and reliable MR results (Sun et al., 2020). We also applied MR-Egger and evaluated its reliability using I^2^ statistics, which check the ‘NO Measurement Error’ assumption (Bowden et al., 2016b).

In addition to the IVs selected from our study, we incorporated public GWAS summary statistics from the Sinnott-Armstrong et al. on HbA1c (Sinnott-Armstrong et al., 2021) with our criteria (see ‘Two-sample MR analysis’ in the Methods section) to select potential IVs for the MR analysis.

To assess the impact of medication use on our primary results, we included individuals who injected insulin or took medications for diabetes (*N* = 318) into the gene-outcome group (*N* = 13,888) to reperform the MR analysis. On the other hand, to eliminate the influence of individuals with T2D, we conducted an additional sensitivity analysis using individuals who were not affected by T2D (gene-exposure: *N* = 273,276; gene-outcome: *N* = 13,304). Moreover, we performed a reverse MR analysis in which we considered adjusted BAG as the exposure and HbA1c as the outcome, to examine the potential causal direction from adjusted BAG to HbA1c (Supplementary Data 2 and Supplementary Data Figure S1). Finally, we investigated the causal effect of HbA1c on regional white matter FA measures to further confirm the causality between HbA1c and adjusted BAG.

## 3 Results

### 3.1 Sample characteristics

The training and testing sets exhibited similar distribution in terms of socio-demographic aspects such as age, sex, and BMI (see more details in Supplementary Figures S2, S3). Similarly, these covariates were evenly distributed among the participants used in the MR study (Table 1).

### 3.2 Estimation of white matter BAG

**Figure 2:**
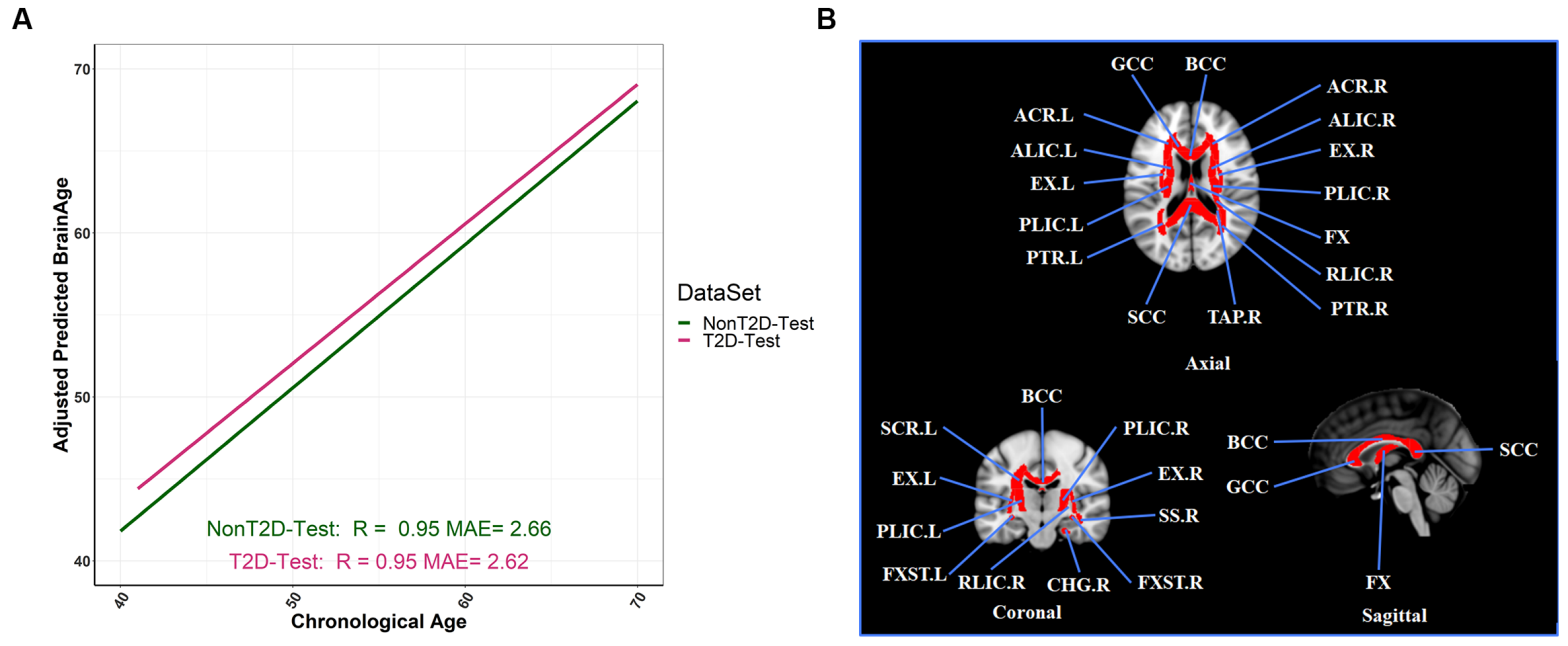
Adjusted BAG and its relationships with T2D status in the testing set. **(A)** The relationship between the adjusted predicted brain age and chronological age in different T2D status (R: coefficients of correlation; MAE: mean absolute error). **(B)** The selection of twenty-six FA tracts from the predictive model for estimation of adjusted BAG, and we colored the FA bundles selected.

Compared with gradient boosting regression and LASSO methods, the RF regression method achieved the best prediction performance in brain age prediction (see Supplementary Table S3), consistent with previous study (Wang et al., 2021). Our optimal RF model attains excellent prediction performance in both the training and testing sets: *R* = 0.97 and MAE (year) = 2.19 for training set (see Supplementary Figure S1); *R* = 0.95 and MAE (year) = 2.66 for non-T2D, and *R* = 0.95 and MAE (year) = 2.62 for T2D in the testing set, respectively (see Figure 2A). A total of 26 FA measures was selected from this optimal model (Figure 2B; see Supplementary Table S3 for their full names and abbreviations). To assess the association (not causal relationship) between BAG and HbA1c, we performed a regression analysis and found that adjusted BAG was significantly associated with HbA1c ( $\hat{\beta }$ = 0.0198; 95% CI = 0.0029, 0.0367; *p*-value = 4.00 × 10^−2^; Supplementary Table S5). Additionally, we observed significant associations between increases in BAG and decline in cognitive function (see Supplementary Table S6).

### 3.3 Two-sample MR analysis

In our study, we selected 842 genetic variants as IVs according to our criteria (see ‘Two-sample MR analysis’ in the Methods section) (see a complete list of IVs in Supplementary Table S7; the Manhattan plot shown in Supplementary Data Figure S2). We identified these IVs that were mapped within previously reported HbA1c-related genes such as *HK1*, *ANK1*, *GCK*, and *CDKAL1* (Leong and Meigs, 2015) using the FAVOR (see Supplementary Table S8).

**Figure 3:**
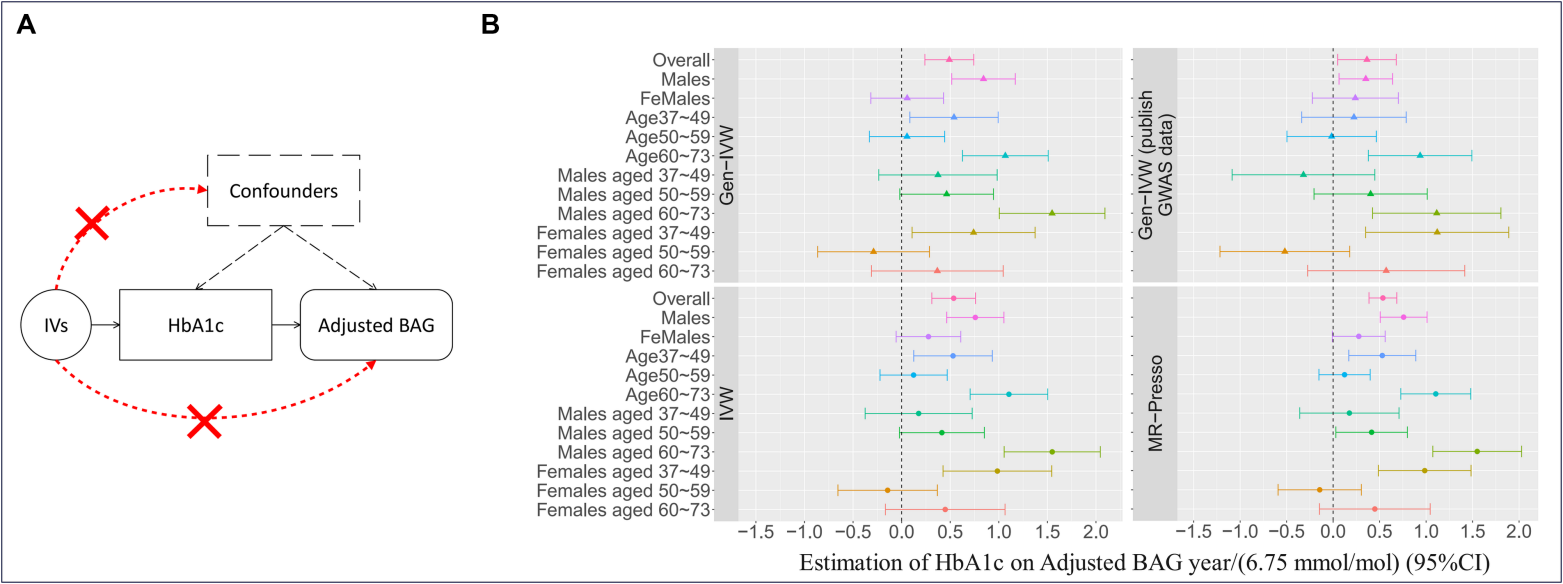
Mendelian randomization and the results. **(A)** The three fundamental instrumental variable (IV) assumptions in the Mendelian randomization (MR) analysis: (I) IVs exhibit a significant association with the exposure (i.e., HbA1c); (II) the exposure is not significantly associated with confounders of the exposure-outcome association, and (III) IVs have an effect on the outcome variable solely through their influence on the exposure. **(B)** The causal effect estimates with a 95% confidence interval (CI) implemented with different MR methods for HbA1c using the IVs selected based on adherence to the three IV assumptions. Gen-IVW (marked with a triangle) is the primary MR method (i.e., weighted generalized linear regression), and the other methods (marked with a dot) are the MR methods used in the sensitivity analysis. Different colors represent different group.

We observed an overall significant and substantial causal effect of HbA1c on adjusted BAG ( $\hat{\beta }$ = 0.49 year/(6.75 mmol/mol); 95% CI = 0.24, 0.74; *p*-value = 1.30 × 10^−4^, as shown in Figure 3) in our two-sample MR analysis. This indicates that an increase in HbA1c levels by 6.75 mmol/mol corresponds to a 0.49-year increase in brain age.

In stratified analyses, we observed that the causal effect of HbA1c on adjusted BAG was particularly prominent in males ( $\hat{\beta }$ = 0.84 year/(6.75 mmol/mol); 95% CI = 0.52, 1.17; *p*-value = 4.32 × 10^−7^) and older individuals (age ≥ 60, $\hat{\beta }$ = 1.07 year/(6.75 mmol/mol); 95% CI = 0.63, 1.51; *p*-value = 2.12 × 10^−6^) (see Table 2 for more details). These results were substantiated by sensitivity analyses using different MR methods (Supplementary Table S9), LOOA (Supplementary Table S10 and Supplementary Figure S4), replication in an independent cohort for IVs selection ( $\hat{\beta }$ = 0.36 year/(6.75 mmol/mol); 95% CI = 0.05, 0.68; *p*-value = 0.02, Supplementary Table S9), inclusion of individuals who administered insulin or utilized diabetes medications ( $\hat{\beta }$ = 0.55 year/(6.75 mmol/mol); 95% CI = 0.32, 0.78; *p*-value = 4.28 × 10^−6^; Supplementary Table S9 and Supplementary Figure S5), and restricting individuals from European ethnic backgrounds without T2D ( $\hat{\beta }$ = 0.50 year/(6.75 mmol/mol); 95% CI = 0.23, 0.77; *p*-value = 2.30 × 10^−4^; Supplementary Table S9).

**Table 2:** The results of two-sample MR analysis between HbA1c and BAG with/without stratification by sex and age.

	Group	Number (gene-exposure; gene-outcome)	MR analysis (gen-IVW)				
			Standardized estimate	Lower (95% CI)	Upper (95% CI)	Estimate (6.75 mmol/mol)	*p* value
*Without stratification (gene-exposure p < 5e-8)*							
HbA1c (842 IVs)	Whole	*N* = (279,011; 13,570)	0.07275129	0.035477893	0.110024687	0.4910712	1.30E-04 ***
*With age stratification*							
HbA1c	37–49	*N* = (70,860; 3,824)	0.079851175	0.012502386	0.147199965	0.5389954	2.01E-02 *
HbA1c	50–59	*N* = (94,467; 5,730)	0.008219022	−0.049138158	0.065576202	0.0554784	7.79E-01
HbA1c	60–73	*N* = (113,684; 4,016)	0.158101865	0.092747876	0.223455853	1.0671876	2.12E-06 ***
*With sex stratification*							
HbA1c	Female	*N* = (156,373; 7,184)	0.008465334	−0.046977127	0.063907794	0.057141	7.65E-01
HbA1c	Male	*N* = (122,638; 6,386)	0.124858402	0.076441673	0.173275131	0.8427942	4.32E-07 ***
*With age by sex stratification*							
HbA1c	37–49 female	*N* = (38,670; 2,139)	0.109703365	0.015898898	0.203507832	0.7404977	2.19E-02 *
HbA1c	50–59 female	*N* = (53,998; 3,136)	−0.042806215	−0.128076578	0.042464148	−0.288942	3.25E-01
HbA1c	60–73 female	*N* = (63,705; 1,909)	0.054434874	−0.046029791	0.154899539	0.3674354	2.88E-01
HbA1c	37–49 male	*N* = (32,190; 1,685)	0.05540717	−0.034840098	0.145654438	0.3739984	2.29E-01
HbA1c	50–59 male	*N* = (40,469; 2,594)	0.068643089	−0.002759449	0.140045626	0.4633409	5.95E-02 ~ .
HbA1c	60–73 male	*N* = (49,979; 2,107)	0.229426417	0.148882193	0.30997064	1.5486283	2.37E-08 ***

~. *p* < 0.1; ^*^p < 0.05; ^**^p < 0.01; ^***^p < 0.001.

We expanded our sample to include individuals from non-European ethnic backgrounds who met our inclusion/exclusion criteria (the gene-exposure group N = 433,374 individuals and the gene-outcome group, N = 14,004 individuals). Within this subgroup, we re-conducted MR analysis, which revealed that elevated HbA1c levels are associated with accelerated white matter brain aging ( $\hat{\beta }$ = 0.29 year/(6.75 mmol/mol); 95% CI = 0.11, 0.47; *p*-value = 1.34 × 10^−3^; see Supplementary Table S9 for more details). Our two-sample reverse MR analysis revealed no significant evidence of a causal effect, indicating the absence of reverse causality in our study (Supplementary Table S11).

In addition, when we altered the outcome variable to regional white matter measures, we found significant causal effects of Hb1Ac on the left hemisphere of posterior corona radiata (PCR-L) ( $\hat{\beta }$ = −0.0259/(6.75 mmol/mol); 95% CI = −0.0289, −0.0229; *p*-value = 1.03 × 10^−65^) and fornix (FX) ( $\hat{\beta }$ = −0.0280/(6.75 mmol/mol); 95% CI = −0.0344, −0.0217; *p*-value = 5.58 × 10^−18^) (see full results in Supplementary Table S12). In our stratified analyses, the causal effects of HbA1c exhibits the most significant impact on FX and anterior corona radiata left (ACR-L) across different age and sex groups. Particularly, these effects were predominantly found in males aged 60–73 years (Supplementary Table S13).

## 4 Discussion

We investigated the causal effects of HbA1c on adjusted BAG by developing innovative computational methods and using large-scale data from the UKBB cohort in this study. We identified significant causal effects of elevated HbA1c on accelerated neurodegeneration during the aging process, as evident by an elevated brain age measured in years. Furthermore, stratified analyses revealed age- and sex-dependent causal effects of HbA1c on adjusted BAG. Specifically, we observed a significantly negative causal effect of HbA1c on adjusted BAG among males in the 60-73-year age group. These findings underscore the importance of glycemic control in preventing and slowing the progression of cognitive impairment.

Our findings are consistent with previous studies (Gudala et al., 2013; Jha et al., 2022). For instance, Jha et al. (2022) reported a 1-unit increase in HbA1c was associated with a 3.88-year increase in the brain age gap. Similarly, we observed that individuals with high levels of HbA1c had elevated adjusted brain age values compared to those with low levels of HbA1c. Numerous studies have found the detrimental effects of elevated HbA1c on neurological health. Diabetes is associated with heightened risks of all types of dementia, and patients with dementia are more likely to be diagnosed with diabetes than patients without dementia (Ohara et al., 2011). Poor glycemic control with elevated HbA1c also increases the risks of diabetic peripheral neuropathies (DPNs) and autonomic neuropathies (Su et al., 2018). These neurological complications are thought to be mediated by various biological factors, including polyol flux, advanced glycation end products (AGEs), oxidative stress, and lipid abnormalities (Tesfaye et al., 2010), suggesting that elevated HbA1c levels may lead to the acceleration of brain aging through these factors. Some study have confirmed that AGEs formed during prolonged hyperglycemia can accumulate in brain tissues and contribute to the pathogenesis of neurodegenerative diseases (Dei et al., 2002; Srikanth et al., 2011). Furthermore, other studies also confirmed that high glucose levels can induce neuronal apoptosis, impair synaptic plasticity, and disrupt the blood–brain barrier, ultimately compromising brain health (Gispen and Biessels, 2000; Pooja Naik, 2014).

To date, there has been no prospective evidence that intensive glycemic control improves cognitive function or prevents cognitive impairment (Launer et al., 2011). However, this may be due to the heterogeneity of the study population, as we found that the impact of HbA1c on brain aging varied widely across different age and sex groups, with a more pronounced effect observed in men and older adults (age ≥ 60). This may be due to the sex differences in energy balance and sex steroids at the molecular, cellular, and tissue levels (Tramunt et al., 2020). Women exhibit specificity in energy partitioning, possibly protecting them from visceral and ectopic fat accumulation (Karastergiou et al., 2013). Such heterogeneity is consistent with prior animal and molecular studies revealing sex differences in glycemic control mechanisms during autonomic nervous system (Takahashi et al., 2020) and clinical neuropathy (The DCCT Research Group, 1988). We provide new insights into how HbA1c contributes to the increased acceleration of brain aging, particularly in older men, and exerts differential effects in a sex-dependent manner, but its causal effects warrant further investigation.

The IVs used in our MR analyses are aligned with previously established diabetes-related genes and add to the robustness of our analysis (Supplementary Table S7 for a complete list). For instance, genes such as *TCF7L2* and *HK1* located on chromosome 10 have been reported to be significantly associated with the risk of diabetes and HbA1c levels, respectively. Similarly, genes *ABCB11* (chromosome 2), *ADCY5* (chromosome 3), *CDKAL1* (chromosome 6), *ANK1* (chromosome 8), *GLIS3* (chromosome 9), and *HKDC1* (chromosome 10) have been linked to various aspects of diabetes, including insulin release, glucose levels, β-cell function, and insulin resistance (Leong and Meigs, 2015; Roman et al., 2017). These existing findings provide support for the validity of our selected IVs and lend credibility to the MR results obtained in our study. In addition, the sensitivity analyses conducted in our study confirmed the absence of substantial horizontal pleiotropy among the selected IVs. This further strengthens the reliability of our MR results, indicating that the estimated causal effects of HbA1c on BAG are unlikely to be confounded by pleiotropic effects.

However, it is important to acknowledge several limitations in our study. Firstly, the individuals recruited from the UKBB may not represent the general population as they are more likely to be “healthy volunteers” living in socioeconomically advantaged areas with a lower prevalence of obesity, smoking, drinking, and other health problems (Fry et al., 2017). This limits the generalizability of our findings and calls for additional research that engages a more diverse population. Secondly, our study focused on a specific age range (37–73 years) and included individuals from a single ethnic background. Thirdly, previous studies have been reported that ethnic differences in T2D associated with genes such as *KCNQ1*, *NOTCH2*, *TCF7L2*, *CDKAL1*, and *KCNJ11* (Waters et al., 2010; Yaghootkar et al., 2020). After incorporating non-European samples into our sensitivity analysis, genetic variants mapped on these genes were selected as IVs. However, gen-IVW cannot address the ethnic-specific effects of IVs, contributing to the difference observed compared to our main findings. Further studies should be incorporated to consider the ethnic-specific effect of IV in the MR approaches. The influence of HbA1c on white matter brain aging may vary across different age groups and ethnicities. Factors such as dynastic effects, assortative mating, social determinants of health, and population stratification could potentially confound the causal relationship between HbA1c and white matter brain aging in different populations (Brumpton et al., 2020; Hwang et al., 2021). Therefore, future studies should aim to replicate our findings in diverse ethnic cohorts to examine the generalizability of the observed causal effects.

## 5 Conclusion

In conclusion, our study provides a comprehensive analysis of the causal relationship between HbA1c and white matter brain aging, demonstrating robust and consistent causal effects using sensitivity analyses. By extending previous associations to causal inference, our findings contribute to a better understanding of the impact of HbA1c on white matter brain aging. Importantly, our study highlights the varying effects of HbA1c on different sex and age groups, emphasizing the significance of blood sugar control strategies to prevent accelerated brain aging in various populations. Nevertheless, future research should address the limitations mentioned above to advance our understanding of the causal effects of HbA1c on brain aging and its implications for preventive strategies.

## Data availability statement

The original contributions presented in the study are included in the article/Supplementary material, further inquiries can be directed to the corresponding authors.

## Ethics statement

The studies involving humans were approved by Ethics Committee/Institutional Review Board: REC reference 21/NW/015. The studies were conducted in accordance with the local legislation and institutional requirements. The participants provided their written informed consent to participate in this study.

## Author contributions

CT: Writing – original draft, Formal analysis. ZY: Writing – original draft, Formal analysis. RM: Writing – review & editing. YP: Writing – review & editing. CB: Writing – review & editing. SG: Writing – review & editing. YM: Writing – review & editing. MC: Writing – review & editing. JY: Writing – review & editing. TL: Writing – review & editing. LH: Writing – review & editing. PK: Writing – review & editing. TM: Writing – review & editing. SC: Writing – review & editing, Project administration, Supervision, Validation. SL: Writing – review & editing, Funding acquisition, Project administration, Supervision, Validation.
