# Supplementary material for: The causal effect of HbA1c on white matter brain aging by two-sample Mendelian randomization analysis
Source: Front Neurosci. 2024 Jan 11;17:1335500. doi: 10.3389/fnins.2023.1335500 (PMC10808780; doi:10.3389/fnins.2023.1335500)
Supplement: Supplementary file 1 [file Data_Sheet_1.DOCX]

Supplementary Material

# Supplementary Data

## **Supplementary Data 1.** Genotype data and summary statistics for the two-sample Mendelian randomization

In our two-sample Mendelian randomization (MR) study, the first sample (gene-exposure group) consisted of 279,011 participants with available HbA1c data but no neuroimaging data. The second sample (gene-outcome group) was non-overlapping of the first sample, which consisted of participants with both HbA1c and neuroimaging data (see Figure 1).

The quality control of UKB genotype data was performed on the first sample following a commonly used procedure . The genotype data came from two platforms, Affymetrix UK BiLEVE Axiom and UKB Axiom® arrays, which captured over 90 million single-nucleotide variants (SNVs) of ∼500,000 subjects . We removed variants with minor allele frequency (MAF) below 0.01, Hardy-Weinberg equilibrium p-value below 0.001, missing genotype rate at 5%, removed participants with more than 2% missing genotypes, and only kept family-unrelated participants with European ethnic backgrounds. We conducted the principal component analysis (PCA) to adjust for population stratification and chose the top 10 principle components (PCs) as recommended by PLINK (version 2.0) and in previous studies (Casanova et al., 2020; Liu et al., 2020; Sofianopoulou et al., 2021).

To obtain the summary statistics for the two-sample MR analysis, we performed the GWAS on the HbA1c (N=279,011) using the first sample, adjusting for age, gender, body mass index, genotyping chip type, and the acquired top 10 PCs using PLINK (version 2.0) . We selected genetic variants with genetic-exposure (HbA1c) p-value < 5×10^-8^.

## **Supplementary Data 2.** Genome-wide association analysis for adjusted brain age gap

We performed reverse causality analysis via MR analysis by treating adjusted BAG as the exposure and HbA1c as the outcome, following the two steps below:

1. Calculated genetic-exposure summary statistics using UKB participants with adjusted BAG data.
2. Calculated genetic-outcome summary statistics using UKB participants with only HbA1c data yet without adjusted BAG data.

IV selection: After pruning and clumping genetic variants, we followed the IV selection introduced in Appendix 3 and selected IVs for MR analysis using criteria below:

- Selected genetic variants with genetic-exposure *p*-value < 1*10^-5^
- Selected genetic variants with genetic-confounder BH*p* > 0.05
- Selected genetic variants with genetic-outcome *p*-value > 0.1

The GWAS summary statistics and annotation of the IVs selected were provided in Supplementary Table 11. The Manhattan plot for GWAS for adjusted BAG is shown in Fig.1 below.

**
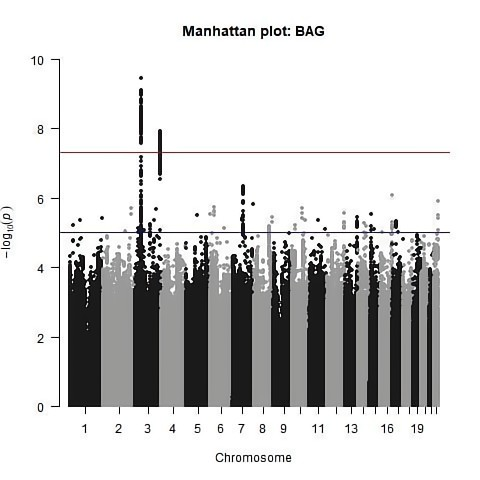
**

**Supplementary Data Figure 1.** Manhattan plot of GWAS for adjusted BAG.

## **Supplementary Data 3.** Manhattan plot of HbA1c.

We provided Manhattan plot of the association analysis for adjusted Brain Age Gap (BAG) at a significance level of 5e-8. More details see Supplementary Table S8.


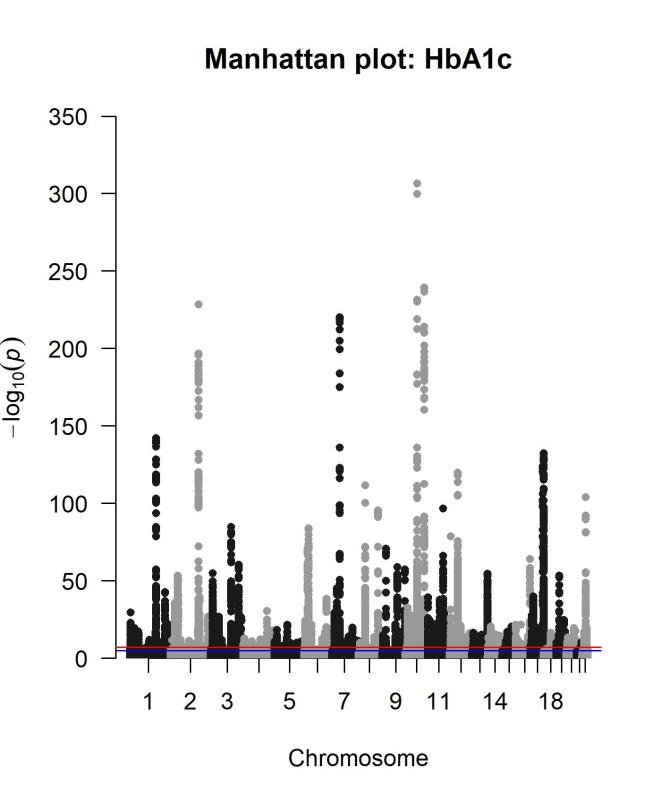


**Supplementary Data Figure 2.** Manhattan plot of GWAS for HbA1c.

# Supplementary Figures

### **Figure S1. Association of predicted brain age.**

A shows the predicted brain age (y-axis) versus chronological age (x-axis) before correction. B shows the predicted brain age after correction. The prediction after the correction revealed improved accuracy according to the increased values of the coefficients of correlation (R) and mean absolute error (MAE (year)). These results indicated that the predicted brain age tended to be overestimated at low chronological age (x-axis) and underestimated at high chronological age (Beheshti et al., 2019; Linli et al., 2022). The prediction can be biased toward the mean when regression minimizes the expected loss function for the optimization procedure (Smith et al., 2019; Butler et al., 2021). This property causes the regression to naturally predicts values toward the mean instead of remaining as extreme as they were initially observed when randomness exists in the sampling process (Beheshti et al., 2019).


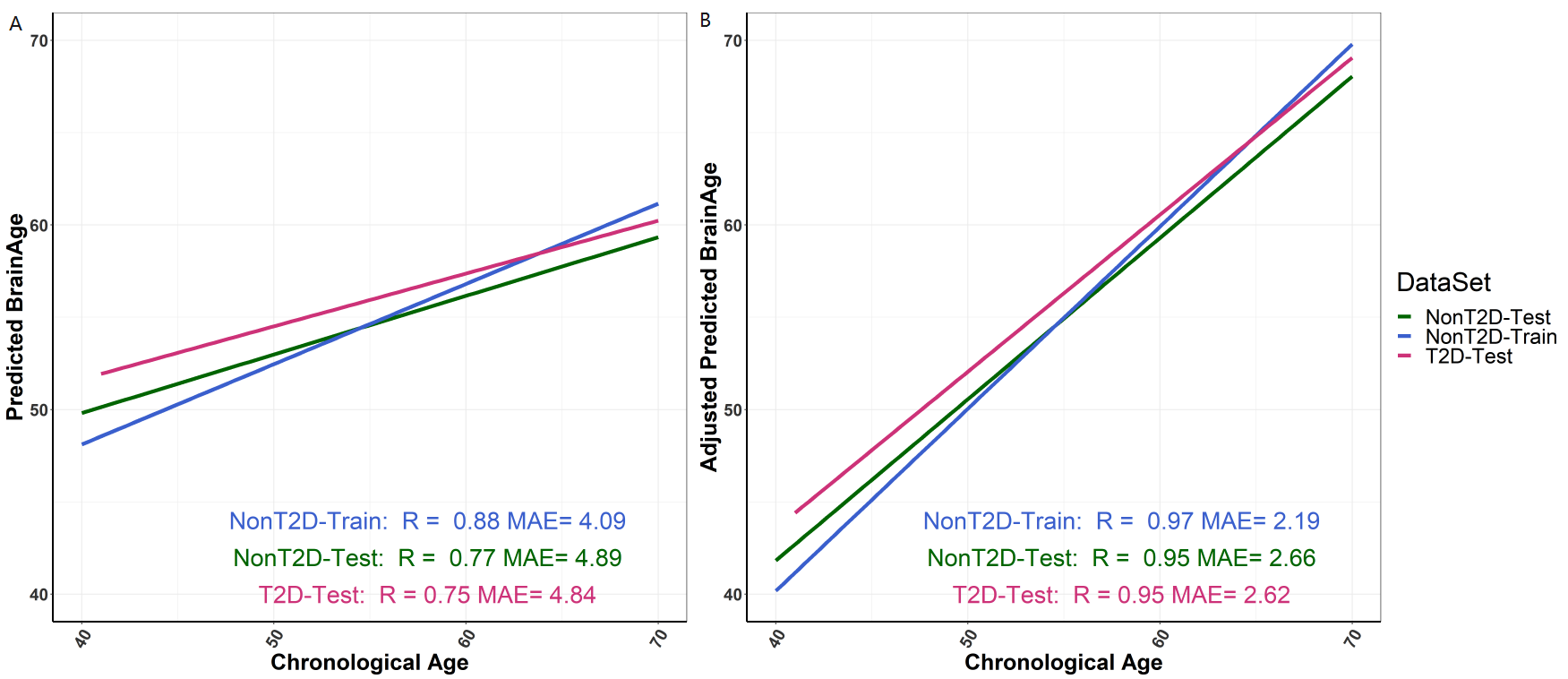


### **Figure S2. The distribution of demographic characteristics for training set and testing set.**

The plots showed characteristics among different sets, including the training sample (non-T2D only) and the study sample ((non-T2D and T2D separately). A and B show the time distribution of age and BMI in the box diagram. C shows the frequency of gender in the bar graph.


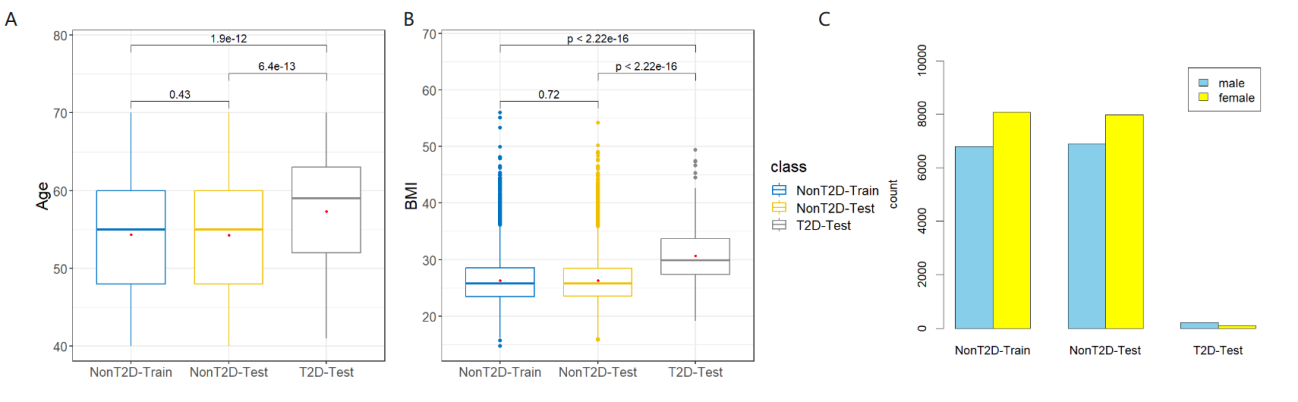


### **Figure S3.** The effect sizes with the 95% confidence interval for testing the difference between non-T2D and T2D within each age category.

**
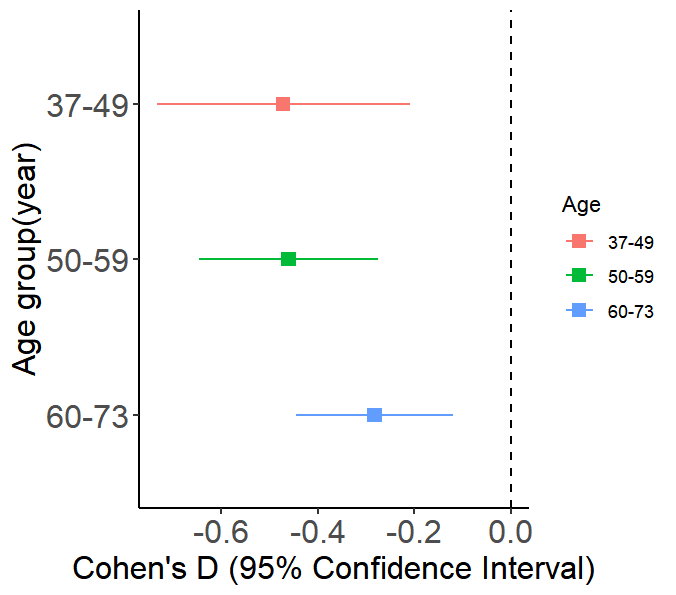
**

### **Figure S4.** Leave-one-out analysis (LOOA) for MR results of Hemoglobin Type A1C (HbA1c). The 10 SNPs from LOOA with the lowest estimated values were selected for plotting. Further details can be found in Supplementary Table S10.

**
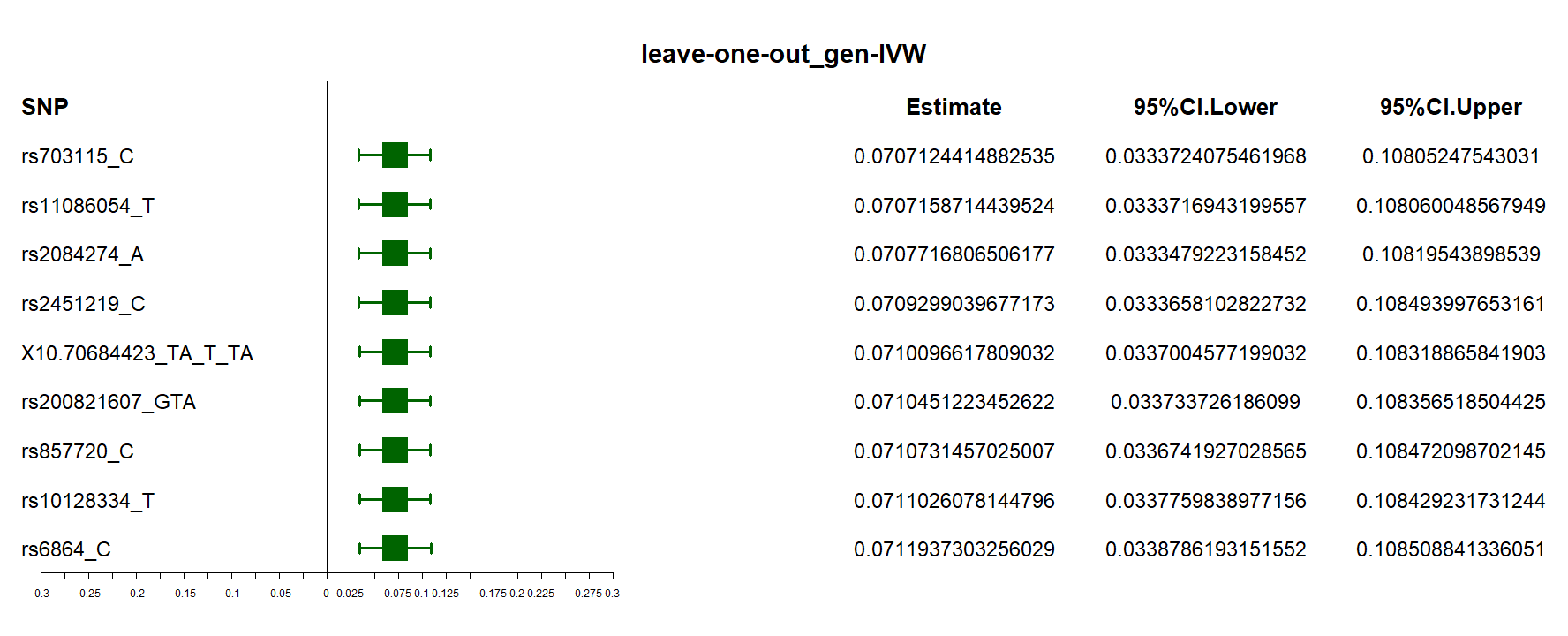
**

### Figure S5. Sensitivity analysis results after including individuals who injected insulin or took medications for diabetes into the gene-outcome group.

The causal effect estimates for HbA1c, along with their corresponding 95% confidence intervals (CI), were obtained using various MR methods that were selected based on adherence to the three instrumental variable (IV) assumptions. The primary MR method, Gen-IVW (indicated by a triangle), represents the weighted generalized linear regression, while the other methods (represented by a dot) were used in the sensitivity analysis. Different colors are used to distinguish between different groups.

**
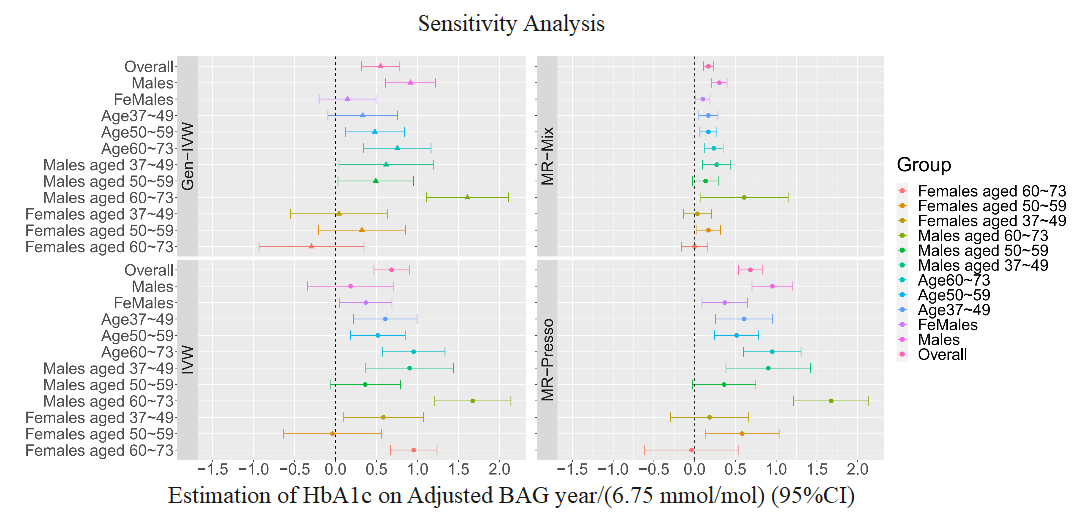
**

**Reference**

Beheshti, I., Nugent, S., Potvin, O., and Duchesne, S. (2019). Bias-adjustment in neuroimaging-based brain age frameworks: A robust scheme. *NeuroImage: Clinical* 24, 102063.

Bergenstal, R. M., Gal, R. L., Connor, C. G., Gubitosi-Klug, R., Kruger, D., Olson, B. A., et al. (2017). Racial Differences in the Relationship of Glucose Concentrations and Hemoglobin A _1c_ Levels. *Ann Intern Med* 167, 95. doi: 10.7326/M16-2596.

Brickman, A. M., Schupf, N., Manly, J. J., Luchsinger, J. A., Andrews, H., Tang, M. X., et al. (2008). Brain Morphology in Older African Americans, Caribbean Hispanics, and Whites From Northern Manhattan. *Arch Neurol* 65. doi: 10.1001/archneur.65.8.1053.

Butler, E. R., Chen, A., Ramadan, R., Le, T. T., Ruparel, K., Moore, T. M., et al. (2021). Pitfalls in brain age analyses. Wiley Online Library.

Casanova, F., Wood, A. R., Yaghootkar, H., Beaumont, R. N., Jones, S. E., Gooding, K. M., et al. (2020). A Mendelian Randomization Study Provides Evidence That Adiposity and Dyslipidemia Lead to Lower Urinary Albumin-to-Creatinine Ratio, a Marker of Microvascular Function. *Diabetes* 69, 1072–1082. doi: 10.2337/db19-0862.

Cavagnolli, G., Pimentel, A. L., Freitas, P. A. C., Gross, J. L., and Camargo, J. L. (2017). Effect of ethnicity on HbA1c levels in individuals without diabetes: Systematic review and meta-analysis. *PLoS ONE* 12, e0171315. doi: 10.1371/journal.pone.0171315.

Linli, Z., Feng, J., Zhao, W., and Guo, S. (2022). Associations between smoking and accelerated brain ageing. *Progress in Neuro-Psychopharmacology and Biological Psychiatry* 113, 110471. doi: 10.1016/j.pnpbp.2021.110471.

Liu, J., Richmond, R. C., Bowden, J., Barry, C., Dashti, H. S., Daghlas, I., et al. (2020). Assessing the causal role of sleep traits on glycated haemoglobin: a Mendelian randomization study. Epidemiology doi: 10.1101/2020.12.18.20224733.

Mayeda, E. R., Karter, A. J., Huang, E. S., Moffet, H. H., Haan, M. N., and Whitmer, R. A. (2014). Racial/Ethnic Differences in Dementia Risk Among Older Type 2 Diabetic Patients: The Diabetes and Aging Study. *Diabetes Care* 37, 1009–1015. doi: 10.2337/dc13-0215.

Selvin, E. (2016). Are There Clinical Implications of Racial Differences in HbA1c? A Difference, to Be a Difference, Must Make a Difference. *Diabetes Care* 39, 1462–1467. doi: 10.2337/dc16-0042.

Smith, S. M., Vidaurre, D., Alfaro-Almagro, F., Nichols, T. E., and Miller, K. L. (2019). Estimation of brain age delta from brain imaging. *Neuroimage* 200, 528–539.

Sofianopoulou, E., Kaptoge, S. K., Afzal, S., Jiang, T., Gill, D., Gundersen, T. E., et al. (2021). Estimating dose-response relationships for vitamin D with coronary heart disease, stroke, and all-cause mortality: observational and Mendelian randomisation analyses. *The Lancet Diabetes & Endocrinology* 9, 837–846. doi: 10.1016/S2213-8587(21)00263-1.

Turney, I. C., Lao, P. J., Rentería, M. A., Igwe, K. C., Berroa, J., Rivera, A., et al. (2023). Brain Aging Among Racially and Ethnically Diverse Middle-Aged and Older Adults. *JAMA Neurol* 80, 73. doi: 10.1001/jamaneurol.2022.3919.

Yousuf, T., Shahid, Q. U. A., Hamdan Tafheem, M. M., Kanwal, M., and Ahmed, A. (2022). Study Finds Racism; Causes Black Americans and Asians Brain to Age Faster than of other Races, Increasing Dementia Risk. *PJMHS* 16, 516–518. doi: 10.53350/pjmhs20221611516.
